# Effects of respiratory exercises in sleep bruxism and associated obstructive sleep apnea: a double-blind, placebo-controlled randomized clinical trial

**DOI:** 10.2340/aos.v83.40252

**Published:** 2024-04-05

**Authors:** Bianca Lopes Cavalcante-Leao, André Luís Porporatti, Rosa Cíntia Felicio Adriano, Rosane Sampaio Santos, Maria Isabel Vanelli, Isabella Perez, Cristiano Miranda de Araújo, José Stechman-Neto, Bianca Simone Zeigelboim

**Affiliations:** a Faculty of Health Sciences, Dentistry, Universidade Tuiuti do Paraná, Curitiba, Paraná, Brazil; b Laboratoire de Neurobiologie Oro-Faciale, Université Paris Cité, Paris, France; c GHPS Assistance Publique Hopitaux de Paris, Paris, France; d Hospital IPO, Instituto Paranaense de Otorrinolaringologia, Curitiba, Paraná, Brazil; e Faculty of Health Sciences, Speech Therapy, Universidade Tuiuti do Paraná, Curitiba, Paraná, Brazil

**Keywords:** Sleep bruxism, positive-pressure breathing, inspiratory, respiratory muscle training, obstructive sleep apnea

## Abstract

**Purpose:**

The aim of this study is to assess the effects of respiratory exercises (inspiratory and expiratory) in individuals with sleep bruxism (SB) and associated obstructive sleep apnea (OSA).

**Methods:**

This is a double-blind, placebo-controlled randomized clinical trial including individuals with SB and associated respiratory events in OSA. Respiratory physical therapy was performed using inspiratory (Threshold® IMT), expiratory (Threshold® PEP) muscle training, and compared with a placebo group. A total of 30 daily respiratory cycles (inspiration and expiration) were performed five times a week for 12 weeks. Individuals were reassessed at two times, at baseline (T1) and after 12 weeks of training (T2) by means of the Pittsburgh Sleep Quality Index and Polysomnography.

**Results:**

Awakening was significantly different (*p* ≤ 0.05) between the inspiratory group and placebo 12 weeks after respiratory physical therapy. The number of contractions of the masseter muscle differed between the inspiratory, expiratory, and placebo groups (*p* ≤ 0.05).

**Conclusion:**

Respiratory physical therapy for OSA improved awaking levels in 80 and 67% of the number of masseter muscle contractions, when compared to placebo.

**Trial registration:**

Brazilian Registry of Clinical Trials (No. RBR-9F6JKM)

## Introduction

Bruxism is a masticatory muscle activity, characterized by repetitive clenching or grinding of the teeth and/or by bracing or thrusting of the mandible, which may have two distinct circadian rhythms, during sleep or during wakefulness [1]. Sleep Bruxism (SB) has recently been targeted as a non-movement disorder or a sleep disorder in healthy individuals [1]. An umbrella review revealed that increased odds for SB were observed with regard to the gastro-esophageal reflux disease, psychotropic medications, genetic polymorphisms, smoking, alcohol intake [2], increasing rhythmic masticatory muscle activity (RMMA) in patients with apnea [3] and a specific type of obstructive sleep apnea (OSA) [4]

Obstructive sleep apnea is a sleep-disordered breathing that consists of recurrent episodes of partial or total obstruction of upper respiratory airways (URA) during sleep, leading to oxyhemoglobin desaturation and sleep fragmentation [5]. Furthermore the percentage of RMMA following respiratory events was significantly higher in OSA patients with SB than in those without SB [4]. It has been discussed that there is an important relationship between OSA and SB, when mild and brief oxygen fluctuations before RMMA may reflect a physiological response that seems to have little influence on SB genesis [6]. However, there was no scientific evidence to support a conclusive relationship between SB and OSA [7].

Obstructive sleep apnea is the most common sleep disorder and a public health concern, with an average prevalence of 34% in men and 17% in women [8]. Tonic muscle contractions, which occurred in SB, can be the cause and effect for the formation of respiratory events. The occurrence of tonic episodes may be the key to understanding the causal relationship between SB and OSA [9]. The most common symptoms are snoring, interrupted normal breathing witnessed by a partner, excessive daytime sleepiness, cognitive alterations (attention and memory deficit), mood changes [10], and impaired quality of life [2]. The standard method to diagnose OSA is a full-night polysomnography (PSG) including measures of oronasal airflow, thoracoabdominal movement, electrocardiogram, pulse oximetry, body position, and snoring intensity [5, 11].

Regarding OSA management, continuous positive airway pressure (CPAP) may be considered a reference standard and consists of generating a positive pressure to maintain the URA open for airflow. However, data related to CPAP adherence are inconsistent and range from 45 to 89% [12]. Another possible management alternative consists of using intraoral devices to project the mandible to an advanced position and prevent pharyngeal collapse. Nevertheless, they are expensive and for some patients can be uncomfortable, which may reduce the adherence and the positive results. Furthermore, physical activity was already proved to be a low-cost, efficient approach to reduce OSA severity and improve sleep efficiency [13]. As proved in another study, if physical activity was coupled with a controlled diet it may reduce the Apnea Hypopnea Index (AHI) and body mass index [14], leading to a better respiratory flow.

Obstructive sleep apnea may also occur by loss of pharyngeal dilator strength that blocks the URA [15]. This loss can be treated using inspiratory and expiratory muscle training, which increases the pharyngeal dilator strength and improves airflow during sleep [16]. Many treatments for respiratory events in OSA are available depending on physiopathology, severity, and individual preference [7]. A systematic review had already reported positive effects of inspiratory and expiratory exercises for OSA [17] and had highlighted their potential as effective treatments with high adherence. Thus, respiratory muscle training may be a simple and low-cost indication of therapy in selected patients.

Even though not fully understood, studies had already contested a possible association between SB and OSA, whereas the presence of SB was higher in OSA patients than in a control group, with an odds ratio of 3.96 [4]. In addition, literature suggests that a better respiratory condition may reduce SB occurrence [7]. Based on these premises, this double-blind, placebo-controlled randomized clinical trial (RCT) aimed to assess the effects of a treatments for respiratory events in OSA, based on inspiratory and expiratory muscle training, in individuals with SB and mild to moderate OSA.

## Methods

The protocol of this study was previously published [18], describing the eligibility criteria and details, and it was registered on the Brazilian Registry of Clinical Trials (No. RBR-9F6JKM, available on ensaiosclinicos.gov.br). This paper was developed according to the Consolidated Standards of Reporting Trials, Extension for Nonpharmacologic Trial criteria [19].

This study consisted of a double-blind, placebo-controlled RCT conducted with subjects with SB and OSA.

Inclusion criteria:

1. Adults between 18 and 65 years old;
2. SB and associated respiratory events (mild to moderate apnea) identified through PSG type 1.

Exclusion criteria:

1. Children and adolescents less than 18 years;
2. Use of intraoral splint;
3. Severe apnea/hypopnea;
4. Severe skeletal abnormalities that affect the upper airways;
5. Chronic medical conditions that affect OSA, such as stroke.
6. Inability to sign the statement of informed consent.

The diagnostic criteria for SB was based on the recommendations of the international consensus for assessment of bruxism through instrumental approaches [1], which makes electromyographic recordings with masseter sensors during sleep. Furthermore, the Pittsburgh Sleep Quality Index (PSQI) questionnaire [20] was applied for OSA and SB symptoms.

For this convenience sample we selected patients who had some signs and symptoms for SB as jaw and neck pain occurs due to the tightening of these muscles during episodes of bruxism. Morning headaches that feel like tension headaches are another potential symptom. Unexplained damage to teeth can also be a sign of nighttime clenching and grinding of teeth as well. Daytime sleepiness can be another probably-related OSA symptom [2].

This diagnosis of OSA was based upon the presence or absence of related symptoms, as well as the frequency of respiratory events during sleep (i.e. apneas, hypopneas, and respiratory effort related arousals) as measured by PSG monitoring. In adults, OSA is confirmed if either of the following two conditions exists:

- There are 15 or more predominant episodes of apnea, hypopnea, or respiratory effort related arousals per hour of sleep (i.e. an AHI or respiratory disturbance index ≥ 15 events per hour) in an asymptomatic patient..
- There are ≥ 5 predominantly obstructive apneas, obstructive hypopneas, or respiratory effort related arousals per hour of sleep (i.e. an AHI or respiratory disturbance index ≥ 5 events per hour) in a patient with symptoms or signs of disturbed sleep and/or the bed partner reports breathing interruptions or habitual snoring and/or the patient wakes with breath-holding, gasping, or choking.

All participants received a respiratory physical therapy, which comprised inspiratory muscle training (IMT) and positive expiratory pressure (PEP) by using the Threshold® instruments. Results were compared with a placebo group.

### Respiratory therapies

Individuals were randomly allocated into inspiratory (IMT), expiratory (PEP), and placebo (non-activated instrument) groups. Maximum respiratory pressures were measured using a manovacuometer that is a pressure gauge.

Inspiratory and expiratory muscle strength was conducted using linear pressure load devices (Threshold® IMT and Threshold® PEP). The training was initiated with a physical therapist verifying the needed resistance by adjusting 70% of the maximum inspiratory or expiratory pressure for treatment groups; no adjustment (0%) was performed for the placebo group. The load was adjusted to a new maximum respiratory pressure every week. A total of 30 daily respiratory cycles (inspiration and expiration) were performed five times a week for 12 weeks.

Individuals were reassessed at two times by the same person at baseline (T1) and after 12 weeks of training (T2) by means of PSQI and PSG. This is a double blind study and for this neither the patients in this study nor the examiner know the group of allocation.

### Inspiratory muscle training

The Threshold® IMT is a device to improve the strength and performance of inspiratory muscles using linear pressure load or independent flow [21]. The device is a transparent plastic cylinder with a mouthpiece on one extremity and a valve closed by the positive pressure of the spring on the other extremity. The valve blocks the airflow until the individual generates enough inspiratory pressure to overcome the pressure of the spring [22].

### Positive expiratory pressure

The Threshold® PEP increases expiratory muscle strength, voice, and speech and may improve swallowing. The device has a closed valve that opens when the pressure generated by the individual is greater than established values [23].

### Randomization, allocation, and blinding

A person not otherwise involved in the study had managed this phase of the study, ensuring that the evaluators of the outcome were blinded to the allocation of the participants to different groups. The subjects were unknown and blinded about the therapy applied. The website program randomization.com was used for the generation of the random sequence. The proportion of the three groups (inspiratory, expiratory, and placebo) was 1:1:1.

The physician responsible for the PSG (AHI, awakenings, and contractions of the masseter muscle) was also blinded.

### Statistical analysis

The normality of the data was assessed using the Shapiro–Wilk test, and the homogeneity of variances was evaluated using Levene’s test. Variables were described using the mean and standard deviation for normally distributed data, and the median and interquartile range (IQR) for non-normally distributed data. The difference between the two assessment moments (before [T1] and after [T2] respiratory therapy) (∆ = T2–T1) was calculated and compared among the three groups. The Kruskal–Wallis test was used, followed by the Dwass–Steel–Critchlow–Fligner post-hoc test. The significance level was set at 5% for all comparisons, and all tests were performed using the Jamovi software (version 1.2).

## Results

The sample comprised 15 individuals allocated into three groups (IMT, PEP, and placebo).

The final sample comprised 13 individuals; two losses were computed due to withdrawal and lack of PSG in the final assessment.

No significant differences were found between the groups during the initial assessment period (T1) (*p* > 0.05) (Table 1).

**Table 1:** Distribution and characteristics of the sample before respiratory therapy (*n* = 15).

	Inspiratory	Expiratory	Placebo	*p* *
**Age**	25.0 (4.84)	35.4 (15.35)	27.6 (5.12)	0.58
**BMI**	27.8 (3.48)	26.16 (5.08)	23.72 (3.12)	0.368
**Initial AHI**	13.54 (6.7)	11.04 (5.78)	7.48(4.44)	0.242
**Initial awakening**	5.6 (1.47)	4.0 (1.06)	3.58 (1.23)	0.058
**Initial contractions of masseter**	3.93 (0.40)	4.3 (1.03)	3.66 (1.55)	0.428
**Initial PSQI**	10 (2.54)	8.8 (3.27)	7.2 (2.16)	0.321

* Kruskal–Wallis test; Average (standard deviation); BMI: body mass index; AHI: hypopnea index; PSQI: Pittsburgh Sleep Quality Index.

**Figure 1:**
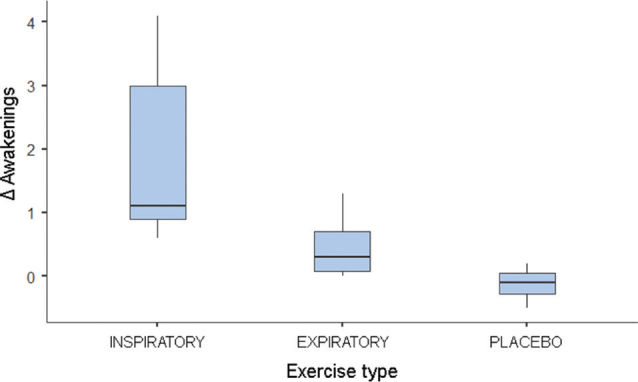
Boxplot of the median of Δ awakenings. ∆ = T2 – T1; T1: assessment before respiratory therapy; T2: assessment after respiratory therapy.

**Figure 2:**
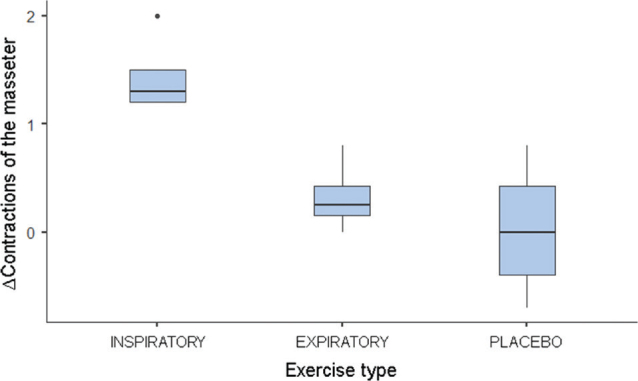
Boxplot of the median of Δ contractions of the masseter muscle. ∆: T2 – T1; T1: assessment before respiratory therapy; T2: assessment after respiratory therapy.

The comparison between T2 and T1 values revealed significant differences in awakening and masseter, with higher values observed in the IMT group for both variables. No differences were found between the groups for the variables PSQI and AHI (Table 2). The post hoc test indicated different Δ awakenings between IMT and placebo (*p* < 0.05; Figure 1). The Δ contraction of the masseter muscle was also different between IMT and PEP (*p* < 0.05) and placebo (*p* < 0.05; Figure 2).

**Table 2:** Differences before and after respiratory therapy (median) (*n* = 13).

	∆ Inspiratory	∆ Expiratory	∆ Placebo	*p* *
	Median (IQR)	Median (IQR)	Median (IQR)	
**PSQI**	0.0 (5.0)	−2.0 (0.75)	0.0 (1.0)	0.327
**AHI**	1.2 (0.7)	0.0 (0.7)	0.0 (0.3)	0.076
**Awakenings**	1.1 (2.1)	0.3 (0.63)	−0.1 (0.22)	0.020*
**Masseter**	1.3 (0.3)	0.2 (0.27)	0.0 (0.82)	0.012*

* Kruskal–Wallis test; ∆: T2 – T1; T1: assessment before respiratory therapy; T2: assessment after respiratory therapy; IQR: Interquartile range; PSQI: Pittsburgh Sleep Quality Index; AHI: apnea and hypopnea index.

## Discussion

This RCT is the first to investigate the effects of inspiratory and expiratory muscle training in individuals with SB and OSA. Our results demonstrated that IMT reduced the number of awakenings (80%) and contractions of the masseter muscle (67%) compared with the placebo group.

According to Akura et al. [4] that is a specific type of OSA in patients with SB and the relationship between RMMA and because of this the purposed inspiratory and expiratory muscle training are limited in improvement in masseter contractions.

Pharyngeal collapse occurs with a tone reduction of the dilator muscle, interacting with anatomic, neural, and functional factors [24]. This association causes a imbalance between the pressure of inspiratory intra-pharyngeal suction and pharyngeal muscle dilators. The OSA etiology also interacts with anatomical-functional factors, such as impaired function of dilator muscles of URA, muscle weakness, and airway anatomy [25].

During a respiratory pause, a cascade of compensatory, hemodynamic, autonomous, and metabolic events restore the haemostatic equilibrium. Then, hemoglobin desaturation (hypoxemia) and carbon dioxide retention (hypercapnia) are detected by peripheral and central chemoreceptors, activating the sympathetic nervous system [6, 26]. The improvement in relation to awakenings and in relation to the number of masseter contractions could be explained by the moment of breathing exercises itself, where the patients reduces their level of activity and seek a moment of self-care [26].

Sympathetic activation also causes micro-arousals that abruptly cease apnea, increasing the activity of pharyngeal muscle dilators and restoring the diameter of URA. Micro-arousals are responsible for sleep fragmentation and excessive diurnal sleepiness [2]. In our study patients who participate in the study in non-placebo intervention have their micro-arousals and RMMA decrease compared to the initial indices. However, the AHI indices do not impact the change in these indices. There was a different view compared to another study that explained the relationship between OSA and SB dependent on the degree of severity of OSA [27].

TMI strengthens the inspiratory muscles, increasing resistance. Although it showed promising results (e.g. better arterial pressure and sleep quality), this exercise has limitations regarding AHI [28]. Nonetheless, TMI is a promising complementary treatment for individuals with OSA and may reduce the contractions of the masseter muscle. OSA is frequently coupled with SB [12, 15] due to oxygen desaturation and excitation reactions [29, 30]. Therefore, treatments for OSA may improve SB-related events.

This study presents important contributions to the field of research; however, it is essential to recognize and consider its limitations, notably related to the restricted sample size. The small size of the studied group may limit the generalization of the results to a wider population, compromising external validity. Furthermore, the representativeness of participants may be affected, as a reduced sample may not adequately reflect the diversity existing in the target population. This limitation implies caution when extrapolating the findings, highlighting the need to replicate the study with a more comprehensive sample to validate and consolidate the conclusions obtained. Despite these restrictions, the study provides valuable insights that can guide future investigations and encourage expanded sampling for a more comprehensive understanding of the phenomenon under study.

There is no scientific evidence that supports a conclusive relationship between BS and OSA; however, studies like this, properly conducted, can elucidate this relationship.

## Conclusion

In this study, we observed that a 12-week IMT improved contractions of the masseter muscle and awakenings, and indicated its potential to treat SB. We suggest new studies to elucidate protocols for IMT implementation as a strategy for treating individuals with SB and OSA.

Regarding application, we suggest combining IMT with other treatments (e.g. CPAP and intraoral devices for mandibular correction) to improve symptoms and efficiently decrease comorbidities.

## Ethical approval

Approval was obtained from the ethics committee of the Universidade Tuiuti do Paraná (n°. 3.684.498). The procedures used in this study adhere to the tenets of the Declaration of Helsinki.

## Informed consent

Informed consent was obtained from all individual participants included in the study.

## Data availability

The datasets generated during and/or analyzed during the current study are available from the corresponding author on reasonable request.

## Conflicts of interest

The authors declare that they have no conflict of interest.

## Authors’ contribution

Conceptualization: Bianca Lopes Cavalcante-Leão, Rosane Sampaio Santos, José Stechman-Neto, Bianca Simone Zeigelboim; Methodology: Bianca Lopes Cavalcante-Leão, Rosane Sampaio Santos, José Stechman-Neto, Cintia Felicio Adriano Rosa, Isabella Perez, Bianca Simone Zeigelboim; Formal analysis and investigation: Bianca Lopes Cavalcante-Leão, Rosane Sampaio Santos, Cintia Felicio Adriano Rosa, Isabella Perez, Cristiano Miranda de Araújo; Writing – original draft preparation: Bianca Lopes Cavalcante-Leão, Cristiano Miranda de Araújo, Bianca Simone Zeigelboim; Writing – review and editing: Bianca Lopes Cavalcante-Leão, André Luís Porporatti, José Stechman-Neto, Cintia Felicio Adriano Rosa, Isabella Perez, Cristiano Miranda de Araújo, Bianca Simone Zeigelboim; Resources: Bianca Lopes Cavalcante-Leão, Cristiano Miranda de Araújo; Supervision: André Luís Porporatti, Bianca Simone Zeigelboim.

## ORCID

Bianca Lopes Cavalcante Leao, https://orcid.org/0000-0002-6170-1914

André Luís Porporatti https://orcid.org/0000-0003-4379-9695

Cíntia Felicio Adriano Rosa https://orcid.org/0000-0002-4885-5775

Rosane Sampaio Santos https://orcid.org/0000-0001-6400-5706

Maria Isabel Vanelli https://orcid.org/0009-0000-9139-2051

Isabella Perez https://orcid.org/0009-0001-7385-589X

Cristiano Miranda de Araújo https://orcid.org/0000-0003-1325-4248

José Stechman-Neto https://orcid.org/0000-0002-0259-2420

Bianca Simone Zeigelboim https://orcid.org/0000-0003-4871-2683
